# Neuroleptic Malignant Syndrome: Diagnostic and Therapeutic Dilemmas

**DOI:** 10.1155/2005/471260

**Published:** 2005-08-04

**Authors:** Abebaw Fekadu, Jonathan I. Bisson

**Affiliations:** ^1^St Ann’s HospitalPooleUK; ^2^University of Wales College of MedicineCardiffUK

## Abstract

Neuroleptic malignant syndrome (NMS) is a life threatening medical state complicating the use of antipsychotic medications and other drugs that affect the dopaminergic system on administration or withdrawal. The condition was recognised nearly half a century ago, shortly after the discovery of antipsychotic medications. However, there are still no systematic studies about NMS. There are no definitive guidelines on its treatment. Although early recognition is emphasised and usually possible, delayed diagnosis is not rare. We here report on a case of NMS complicated by renal failure, and possibly respiratory failure. The report underscores the seriousness of delayed diagnosis and puts forward a comprehensive management recommendation based on our experience and the existing literature.

## 1. Introduction

NMS has been recognised since the introduction of haloperidol [2,4], and affects up to 1.4% of those treated with antipsychotic medications [1,17]. It is reported from most parts of the world and in all age groups [11, 12,16,22,28]. It is typically associated with the old generation (typical) antipsychotic medications. However, there are several reports of NMS on almost all new generation (atypical) antipsychotic medications including clozapine [6,9,13,14,19,21,24,29]. The clinical manifestations of NMS are primarily neuromuscular. According to the Diagnostic and Statistical Manual of Mental Disorders fourth Edition (DSMIV) [1], the development of muscular rigidity and fever associated with the use of antipsychotic drugs are the central features of NMS. DSM-IV further requires at least two more features from a list of ten, which are mainly secondary to autonomic instability and extrapyramidal side effects. These include diaphoresis, tachycardia, elevated or labile blood pressure, dysphagia, tremor, incontinence, changes in level of consciousness ranging from confusion to coma, mutism, leucocytosis and laboratory evidence of muscular destruction (e.g. elevated creatinine phosphokinase). Diagnosis assumes exclusion of general medical conditions and mental disorders that could explain the symptoms. The presentation of NMS is similar with most antipsychotic medications including atypical antipsychotics [13]. However, fever and rigidity are reported to be less prominent with clozapine induced NMS [9]. The DSM-IV does not accommodate for these differences.

Although several risk factors are identified [4,25], the actual cause is undetermined and interest has focused on biochemical mediators and genetic factors [7, 10,11,23,26]. A mortality rate of 20% is reported to be associated with NMS [11]. A more careful analysis of data has indicated a pattern of decline in mortality [12,20] through the years, but still substantial mortality remains [20]. The primary treatment of NMS is withdrawal of medication considered to be causative. Once medication is withdrawn, recovery usually occurs within two weeks, although there is a suggestion that some residual symptoms may last from several weeks to months [5]. Patients will usually require antipsychotic medication within or shortly after the period of recovery. Time elapsed after a resolution of NMS was identified to be the key factor to prevent relapse of NMS on reintroduction of antipsychotics. Reintroduction of antipsychotic medication within two weeks of resolu-

ISSN 0953-4180/05/$17.00 © 2005 – IOS Press and the authors. All rights reserved tion of an NMS episode had resulted in immediate relapse [19]. Therefore the general recommendation is to reintroduce antipsychotic medication after two weeks of resolution of symptoms.

Diagnosis is generally clinical, and early recognition can be life saving; however, diagnosis and treatment are not always straightforward. This article attempts to illustrate the diagnostic and therapeutic challenges of NMS in general medical settings based on a case report. The article also suggests comprehensive steps, based on the literature and our own experience, which should be considered for the early detection and management of NMS.

## 2. Case report

Mr A was a 45-year-old patient with a diagnosis of paranoid schizophrenia made when he was 20 years of age. At the same time, a mild learning disability (with IQ of 60) was identified. Compliance with treatment and engagement had always been difficult. He required depot antipsychotic maintenance injections from early on. He was on fluphenazine decanoate initially, and later flupenthixol decanoate. Eighteen months before admission to hospital for acute bilateral pneumonia and subsequent NMS symptoms, he was initiated on zuclopenthixol decanoate, and the dose was progressively increased to 500 mg weekly four months prior to admission. The acute pneumonia shortly evolved into respiratory failure, and later renal failure. The acute renal failure was subsequently deemed to have been caused by a massive myoglobinuria. Intensive medical treatment including haemodialysis was provided for 10 days; however, he continued having hyperpyrexia, labile blood pressure and tachycardia.

During psychiatric review, Mr A was profusely sweating, had moderately severe generalized lead pipe rigidity, and was disorientated, repeatedly trying to pick on his dressed wound. He had a grossly raised urine myoglobin (10758 ng/ml) and a moderately elevated serum creatine kinase (CK) (992 IU/l). NMS was considered a possible diagnosis and his depot zuclopenthixol decanoate was withheld. After that the fever and autonomic instability improved quickly as did his level of consciousness. Within two weeks, the rigidity subsided. Four weeks after his last depot medication, his CK had decreased to 51 IU/l.

Because of a history of difficult to control paranoid symptoms it was believed to be important to recommence antipsychotic medication although there was no evidence of a deterioration of Mr A's chronic residual psychotic symptoms whilst antipsychotic medication was withheld. Risperidone 1 mg once a day was commenced after the resolution of the extrapyramidal symptoms. The dose of the risperidone was gradually increased to 4 mg twice a day after discharge. Six months after the episode he had not shown signs of recurrence of NMS and was compliant with the risperidone. His mental state, creatine kinase and renal function tests have all remained stable.

## 3. Discussion

Mr A exhibited most of the clinical features of NMS and satisfied the diagnostic criteria specified in the DSM IV. Despite these clinical and laboratory indicators actual diagnosis of NMS was difficult with Mr A. This stemmed from three main challenges. Firstly, most diagnostic criteria require exclusion of physical causes that could explain the symptoms of a possible NMS. Mr A initially had pneumonia. This meant NMS was not considered until raised CK, and myoglobinuria, an indicator of rhabdomyolysis, were detected. The presence of rhabdomyolysis is not essential in the diagnosis of NMS according to the DSM-IV criteria. This seems reasonable considering the fact that rhabdomyolysis is non-specific and also rare. However, in the case of Mr A. presence of evidence of rhabdomyolysis of no other detectable cause made it easier to make a putative diagnosis of NMS. Excluding other possible physical causes of NMS-like symptoms is essential. However, given the morbidity and mortality associated with NMS it is crucial to exercise considerable caution before discounting NMS as a possibility. Secondly, NMS is usually expected to start abruptly in the first two to four weeks after initiation of antipsychotic medications or shortly after incrementing the dose of antipsychotics [1]. In the case of Mr A, he was on the flupenthixol decanoate depot for over a year and the last dose increment happened four months prior to onset of NMS symptoms. This delay in onset was confusing. Thirdly, NMS can be complicated by other physical conditions that hide the symptoms of NMS that could further delay recognition. It is possible that Mr A had developed NMS that was later complicated by the occurrence of serious medical conditions, i.e., pneumonia, and renal and respiratory failure. It might also be possible that he initially developed pneumonia that predisposed him to develop NMS followed by other serious complications.

The challenges in this case typify the challenges encountered in detecting and treating NMS in medical settings. Minimising the possibility of physical illness in favour of NMS can have a dire consequence. On the other hand, minimising the possibility of NMS in favour of a physical condition can also have serious consequences, as the case here seems to illustrate. It can also be tempting to stop antipsychotic medications in many serious medical conditions. This is unlikely to be a safe practice. Medical illness is one of the risk factors for relapse of a psychotic illness. Therefore, stopping antipsychotic medication could mean unnecessary suffering for the patient. Getting the full participation of the patient in the treatment of the medical state can also be very difficult. A further ramification that can be demonstrated in this case is the challenge of finding a safe depot alternative once a patient has developed NMS to one conventional antipsychotic depot. Although all antipsychotics are associated with NMS, this risk is believed to be lesser with atypical antipsychotics. In Mr A's case, we opted to start with risperidone, which is the only atypical antipsychotic medication with depot option.

Definitive treatment recommendations of NMS are conspicuous by their absence. Based on our experience and the literature we suggest 11 steps for the management of NMS. These suggestions are not meant to be definitive. However, we believe these are the minimum standards that should be considered in the management of NMS.

Recommended steps for consideration in the early detection and management of NMS

1. Consider NMS in a patient presenting with fever, rigidity or clouding of sensorium, and taking any antipsychotic medication or a medication that could adversely affect the dopamine system such as metoclopromide, and L-dopa and amantadine (on withdrawal). Monitor autonomic functions and check for laboratory indicators such as CK, leucocytosis, raised ESR and possibly myoglobinuria. With atypical antipsychotics the presentation might be with change in the nature of behavioural symptoms or clouding of sensorium rather than with the typical core symptoms of fever or rigidity.
2. Confirm diagnosis using a standard diagnostic criterion, such as DSM-IV. We do acknowledge the limited knowledge we have about the validity of this criterion for NMS. However, it appears to have face validity and to have a reasonably low threshold to detect NMS. Such a low threshold is particularly appealing in the diagnosis of such life threatening states.
3. When a strong suspicion or evidence of NMS exists follow steps 4–10. If detected early, NMS can run a benign course. However, admission to a medical unit will usually be indicated. With early detection, some milder cases can be managed in non-medical settings, for example on a psychiatric ward, as long as there is close monitoring and good medical cover.
4. Stop antipsychotic medication immediately. In most cases antipsychotics can be stopped safely. There is not enough information on the course and best management practice on NMS symptoms related to withdrawal of dopamine agonists. If symptoms are serious restarting withdrawn medication may be helpful as long as it can be done safely.
5. Monitor vital functions such as blood pressure, pulse, temperature and respiration.
6. Serially monitor CK. Normally the level declines quickly once the antipsychotic is stopped.
7. Attend to hydration, nutrition, renal function, concomitant infection, and other medical complications.
8. There is no specific medication for NMS. Benzodiazepines, anticholinergics, muscle relaxants [6] and dopamine agonists [29] can be used depending on severity of symptoms. Medical complications such as renal and respiratory failure need aggressive treatment in the usual way.
9. Consider using heparin to prevent venous thrombosis and pulmonary embolism [15].
10. Reintroduce antipsychotics cautiously after two weeks of resolution of symptoms of NMS [18, 19]. Reintroduction before that period is associated with increased risk of relapse of NMS. Use atypical or low potency antipsychotics and increase gradually. If active psychotic symptoms start soon after withdrawal of antipsychotic medication, commencing benzodiazepines may be helpful. If this fails to work at higher dose or if symptoms are very severe, ECT may be considered [27]. Monitor CK in the initial phases of reintroduction of antipsychotics. When depot antipsychotics are indicated, start with oral medications. As the only atypical antipsychotic with a depot option, risperidone might be a good choice.
11. NMS can recur at any time. The long-term management plan should alert those involved in ongoing care to this.

## 4. Conclusion

NMS will probably remain a clinical diagnosis based on a high index of suspicion for several years to come. It is more frequent than is acknowledged and should not be considered a diagnosis of exclusion. With the increasing diversion of provision of psychiatric service to primary and community based models, it is imperative that NMS is recognised at all levels. It has to be emphasised that NMS that is not detected at its early stage is serious and costly. In order to ensure timely and appropriate management of NMS, reasonable steps such as those suggested above should be adopted and followed.

## Acknowledgements

We are grateful to Mr A for his permission to report his presentation, Dr Riley for his contribution to the physical facts and Drs Mirando and Ali, and Mr Smith for providing information relating to the ongoing psychiatric management of Mr A.
